# Opening New Gates for the Modification of PVC or Other PVC Derivatives: Synthetic Strategies for the Covalent Binding of Molecules to PVC

**DOI:** 10.3390/polym8040152

**Published:** 2016-04-19

**Authors:** Rodrigo Navarro, Mónica Pérez Perrino, Carolina García, Carlos Elvira, Alberto Gallardo, Helmut Reinecke

**Affiliations:** Instituto de Ciencia y Tecnología de Polímeros (ICTP-CSIC), Calle Juan de la Cierva 3, 28863 Madrid, Spain; rnavarrocrespo@gmail.com (R.N.); cruzdp@yahoo.es (M.P.P.); carolina@ictp.csic.es (C.G.); celvira@ictp.csic.es (C.E.); gallardo@ictp.csic.es (A.G.)

**Keywords:** PVC, chemical modification, migration, trichlorotrizaine chemistry

## Abstract

Several synthetic strategies based on the use of substituted aromatic and hetero-aromatic thiols for the covalent binding of modifier compounds to PVC are described. A variety of aliphatic alcohols and amines are linked to the aromatic or heteroaromatic rings via highly active functionalities as the isocyanate, acidchloride, or chlorosulfonyl group, and the three chlorine atoms of trichlorotriazine. The first three pathways lead to protected aromatic disulfides obtaining the substituted aromatic thiols by reduction as a final step of an unprecedented synthetic route. The second approach, in a novel, extremely efficient, and scalable process, uses the particular selectivity of trichlorotriazine to connect aliphatic amines, alcohols, and thiols to the ring and creates the thiol via nucleophilic substitution of a heteroaromatic halogen by thiourea and subsequent hydrolysis. Most of the modifier compounds were linked to the polymer chains with high degrees of anchorage. The presented approaches are highly versatile as different activations of aromatic and heteroaromatic rings are used. Therefore, many types of tailored functional nucleophiles may be anchored to PVC providing non-migrating materials with a broad range of applications and properties.

## 1. Introduction

Poly(vinyl chloride), PVC, is one of the most versatile plastics of the modern world and places second in the market share of polymeric materials [1,2]. This is principally due to its high versatility and excellent balance between low production costs and general properties. Around 35% of PVC produced in the world is used for the production of soft-PVC articles which contain large amounts of plasticizers yielding inexpensive articles useful for applications in the cables and films industry, as children toys, kitchen floors, or biomedical devices, such as blood bags, catheters, protective gloves, *etc*.

In many of these applications, migration of the plasticizer from the PVC item towards the medium in contact may occur [3]. Migration depends on the nature and amount of the plasticizer, the plasticizer’s structure, its compatibility with the polymer, and the temperature. In any case, this leaching of the additive leads to a progressive loss of the initial properties and implies serious health hazards when PVC articles for biomedical applications or children toys are dealt with because additives migrated to the article’s surface can contaminate physiological fluids, like blood, serum, or plasma [4,5]. In particular, dioctyl phthalate (DOP), one of the most frequently used plasticizers for PVC, was shown to produce toxic and adverse effects, especially in animal or human tissues, such as the pituitary gland, liver, or testicles [6,7]. For this reason, DOP was banned a few years ago for certain applications by the U.S. Consumer Product Safety Commision (CPSC).

Many different strategies [8,9,10,11,12,13,14,15] have been proposed to solve this migration problem, one of them being the covalent binding of the plasticizer to the PVC chains [16,17,18,19,20]. This binding reaction can be carried out by simple nucleophilic substitution of the chlorine atoms. The most frequently used nucleophiles for the chemical modification of PVC have been azide, thiolates, thiocyanate, hydroxide, iodide, inorganic sulfides, and phthalimides. An excellent review article about this subject was recently published by Moulay [21]. Particularly efficient, due to their strong nucleophilicity and simultaneously low basicity, are azides and aromatic thiols. Azide groups, for example, have recently been shown to undergo copper-free click reactions with alkynes, a process that allowed for the covalent linkage of long aliphatic plasticizer molecules to the polymer [16]. The same goal was reached using aromatic thiol compounds that were modified in ortho or para positions of the aromatic ring [17,18,19,20]. The main drawback of all these attempts to prepare covalently-bound PVC-plasticizer systems was that they were industrially non-viable. In order to be successful in this aspect the chosen chemistry should be economically and ecologically viable and, furthermore, fulfill the following conditions: easy to prepare, quantitative linkage to the polymer, and high plasticizer efficiency.

In this article we present and analyze novel synthetic approaches based on the use of aromatic and hetero-aromatic thiols for the covalent linkage of model molecules to PVC. The aspect of the plasticizer efficiency will be dealt with in a future paper which will be dedicated to a systematic study of the influence of the chemical structure, chain length, and plasticizer-PVC compatibility on the glass temperature of the modified polymer.

## 2. Materials and Methods

Commercial bulk polymerized PVC was obtained from Rio Rodano Industries, Spain. The average molecular weights determined by GPC were *M*_W_ = 112,000 g/mol and *M*_N_ = 48,000 g/mol. The tacticity measured by ^13^C–NMR was syndio = 30.6%, hetero = 49.8% and iso = 19.6%.

2-Mercaptobenzoic acid, 4-mercaptobenzoic acid, 2-aminothiophenol, 4-aminothiophenol, and diphenyldisulfide were purchased from Acros (Acros, Madrid, Spain) or Sigma (Sigma, Madrid, Spain) with purities higher than 96%. TCTA, bis(2-ethylhexyl)amine, bis(*n*-butyl)amine, 2-ethylhexan-1-ol, and 2-ethylhexane-1-thiol used were also from Sigma and had a purity of 99%.

^1^H-NMR and IR analysis. ^1^H-NMR spectra were recorded at 25 °C in 5% (*w*/*v*) CDCl_3_ or DMSO-d solutions with a Varian Gemini 300 MHz under standard conditions. IR measurements were performed on a Perkin Elmer Spectrum One IR-Spectrophotometer (Perkin Elmer, Madrid, Spain).

Preparation of **1a**, **1b**, **3a**, **3b**: the aromatic mercapto compound was dissolved in an ethanol/water mixture and stoichiometric amounts of hydrogen peroxide were added at room temperature together with catalytic amounts of ammonia. After stirring the reaction mixture for two hours, methylene chloride was added, the organic phases washed twice with water and dried with magnesium sulfate. After elimination of the solvent the products were obtained in yields higher than 90%.

Preparation of acid chlorides **2a** and **2b**: disulfide **1a** or **1b** was heated to reflux for 2 h with a five-fold excess of thionyl chloride and catalytic amounts of dimethylformide (DMF). Excess solvent was then distilled off and the oily residue stirred in hexane until crystallization took place. Filtration and drying yielded yellow needles of melting points 152 °C (for the ortho derivative) and 82 °C for the para derivative. Both data correspond well to data found in the literature [22,23] **2a**: ^1^H–NMR (DMSO-d6) 8.02 (d, 2H), 7.62 (d, 2H), 7.56 (t, 2H), 7.34 (t, 2H; **2b**: ^1^H–NMR (CDCl_3_) *δ*(ppm); 8.05 (d, 4H), 7.59 (d, 4H).

Preparation of isocyanates **4a** and **4b**: 1 mol disulfide **3a** or **3b** was dissolved together with a four-fold molar amount of triethylamine in methylene chloride and cooled to 0 °C under a flux of nitrogen. 0.67 mol triphosgene dissolved in methylene chloride were then added to the cold mixture. After five minutes stirring the reaction was finished and the mixture was washed twice with ice cold water. After drying the organic phase the solvent was stripped off. The diisocyanate was obtained, in the case of the para derivative, as a stable yellow crystalline substance of melting point 85 °C and in the case of the ortho derivative as yellow powder that tends to get grey-green at higher temperatures (and should, therefore, be stored at a cold and dry place). Yield: 93%, ^1^H–NMR (CDCl_3_) of the ortho-product: δ 7.44 (d, 2H), 7.30 (t, 2H), 7.13 (d, t, 4H). ^1^H-NMR (CDCl_3_) of the para-product: δ 7.42 (d, 4H), 7.02 (d, 4H).

Preparation of disulfonylchloride **5**: 0.1 mol diphenyldisulfide dissolved in 100 mL chloroform was added to a solution of 0.3 mol chlorosulfonic acid in chloroform so that the temperature did not exceed 10 °C. The mixture was then stirred for one hour at 40 °C. After quenching on ice, the phases were separated, the organic phase washed twice with sodium bicarbonate solution and dried with magnesium sulfate. The solvent was eliminated and the product obtained as yellow powder with a yield of 70%. ^1^H–NMR (CDCI_3_): δ 7.75 (d, 4H), 8.05 (d, 4H).

Preparation of **oE-di** and **pE-di**: **2a** or **2b** were dissolved in methylene chloride and cooled to 0 °C. Then, a solution of equimolar amounts of alcohol and triethylamine in methylene chloride was slowly added. After one hour stirring at 40 °C the mixture was cooled, washed twice with water, and dried with magnesium sulfate. After elimination of the solvent, the esters were obtained with yields around 90%. ^1^H–NMR (CDCl_3_) of **oE-di**: δ 8.05 ppm (d, 2H), 7.75 (d, 2H), 7.38 (t, 2H), 7.20 (t, 2H), 4.32 (d, 4H), 1.76 (m, 2H), 1.55–1.25 (m, 16H), 0.96 (t, 6H), 0.90 (t, 6H). ^1^H–NMR (CDCl_3_) of **pE-di**: δ 7.97 (d, 4H), 7.52 (d, 4H), 4.22 (d, 4H), 1.76 (m, 2H), 1.55–1.25 (m, 16H), 0.96 (t, 6H), 0.90 (t, 6H).

Preparation of **oA-di** and **pA-di**: **2a** or **2b** were dissolved in methylene chloride and cooled to 0 °C. Then, a solution of equimolar amounts of amine in methylene chloride was slowly added. After one hour stirring at 40 °C the mixture was cooled, washed twice with water, and dried with magnesium sulfate. After elimination of the solvent the amides were obtained with yields of 96%. ^1^H–NMR (DMSO-6d) of **oA-di**: 7.66 (d, 2H), 7.44–7.30 (t, t, 4H), 7.24 (d, 2H), 3.39 and 3.07 (s, 4H), 1.76 (m, 2H), 1.50–0.56 (m, 28H). **pA-di** (CDCl_3_): δ 7.48 (d, 4H), 7.30 (d, 4H), 3.41 (m, 2H), 3.16 (d, 2H), 1.78 (m, 2H), 1.55–0.85 (m, 16H), 0.84 (t, 6H), 0.68 (t, 6H).

Preparation of **SE-di** and **SA-di**: disulfonyl chloride **5** was dissolved in chloroform and equimolar amounts of 2-ethylhexyl alcohol (in the case of **SE-di**) and bis-(2-ethylhexyl)amine (in the case of **SA-di**) and triethylamine were added. The mixtures were stirred for 20 h at 60 °C. The solutions were washed twice with water, dried, and the solvent eliminated. **SA-di** was obtained with a yield of 95% and was used for the reduction step without purification, **SE-di** required purification by means of column chromatography (on silica gel as stationary phase and methylene chloride as solvent) and was finally obtained with a yield of 36%. ^1^H–NMR (CDCl3) of **SA-di**: 7.72 (d, 4H), 7.57 (d, 4H), 2.93 (m, 4H), 1.55 (m, 2H), 1.39–1.13 (m, 16H), 0.86 (t, 6H), 0.81 (dt, 6H). H–NMR (CDCl3) of **SE-di**: 7.76 (d, 4H), 7.59 (d, 4H), 3.88 (d, 4H), 1.47 (m, 2H), 1.30–1.00 (m, 16H), 0.80–0.65 (m, 12H).

Preparation of **oU-di** and **pU-di**: **4a** or **4b** were dissolved in methylene chloride and cooled to 0 °C. Then, a solution of equimolar amounts of amine and catalytic amounts of stannous octoate in methylene chloride was slowly added. After 6 h stirring at 40 °C the solvent was eliminated and the residue subjected to the reduction step without further purification. ^1^H–NMR (CHCl_3_) of **oU-di**: 8.18 (d, 2H), 7.54 (t, 2H), 7.38 (t, 2H), 7.22 (d, 2H), 6.91 (s, 2H), 4.05 (d, 4H), 1.63 (m, 2H), 1.32 (m, 16H), 0.92 (m, 12H). **pU-di** (CDCl_3_): δ 7.42 (d, 4H), 7.32 (d, 4H), 6.69 (s, 2H), 4.09 (dd, 4H), 1.60 (m, 2H), 1.45–1.25 (m, 16H), 0.91 (m, 12H).

Preparation of **oH-di** and **pH-di**: analogue to the previous procedure. However, the reactions were finished after only 30 min. ^1^H-NMR (CHCl_3_) of **oH-di**: 8.40 (s, 2H), 7.72 (t, 2H), 7.38 (t, 2H), 7.07 (d, 2H), 6.81 (s, 2H), 3.18 (dd, 4H), 1.79 (m, 2H), 1.31 (m, 16H), 0.92 (m, 12H). **pH-di** (CDCl_3_): δ 7.39 (d, 4H), 7.31 (d, 4H), 6.36 (s, 2H), 3.22 (d, 4H), 1.70 (m, 2H), 1.45–1.25 (m, 16H), 0.91 (m, 12H).

Reduction of the disulfides: The disulfides were dissolved in a 1:1 mixture of THF and isopropanol and 2 eq of NaBH_4_ were added. After stirring for 15 h at room temperature, 1 N HCl is added until the pH of the mixture is 1–2. Then, the product was extracted with methylene chloride, dried over magnesium sulfate, and the solvents eliminated. The obtained thiols were used for the next step without further purification: the yields were higher than 95% in all cases.

Modification of PVC with thiols prepared by approach one: in order to preform the thiolate sodium salt, the thiol was dissolved under nitrogen in a small quantity of dry ethanol. Then, an equimolar amount of NaOH powder was added and the solution stirred for 15 min at room temperature until a clear solution was obtained. The required amount of this solution was added to a sealable flask which contained PVC previously dissolved under a flow of nitrogen in a 4:1 mixture of Acetone/THF. The reaction mixture was heated to 85 °C for 2 h and was then precipitated in cold methanol. Purification was achieved using three solution-precipitation cycles in THF/methanol.

**oE** (in DMSO-d): 7.88 (d, 1H), 7.52 (d, 1H), 7.37 (t, 1H), 7.18 (t, 1H), 5.26 (s, SH), 4.16 (d, 2H), 1.62 (m, 1H), 1.45–1.15 (m, 8H), 0.85 (t, 3H), 0.82 (t, 3H).

**pE** (in CDCl_3_): 7.79 (d, 2H), 7.29 (d, 2H), 4.23 (d, 2H), 3.61, (s, SH), 1.71 (m, 1H), 1.55–1.25 (m, 8H), 0.94 (t, 3H), 0.90 (t, 3H).

**oA** (in CDCl_3_): 7.28 (d, 1H), 7.20–7.10 (m, 3H), 3.62 (s, SH), 3.39 and 3.07 (s, 2H), 1.76 (m, 1H), 1.50–0.56 (m, 14H).

**pA** (in DMSO-d): 7.30 (d, 2H), 7.15 (d, 2H), 5.64 (s, SH), 3.45 and 3.12 (s, 2H), 1.8–1.4 (m, 1H), 1.4–0.90 (m, 8H), 0.90–0.55 (m, 6H).

**oU** (in CDCl3): 8.18 (d, 1H), 7.54 (t, 1H), 7.38 (t, 1H), 7.22 (d, 1H), 6.91 (s, 1H), 4.05 (d, 2H), 3.63 (s, SH), 1.63 (m, 1H), 1.32 (m, 8H), 0.92 (m, 6H).

**pU**: 7.45–7.23 (m, 4H), 6.68–6.53 (m, 1NH), 4.08 (d, 2H), 3.41 (s, 1SH), 1.60 (m, 1H), 1.45–1.25 (m, 8H), 0.97–0.85 (m, 6H).

**oH** (in CDCl3): 8.17 (d, 1H), 7.48 (m, 2H), 7.28 (t, 1H), 6.91 (t, 1H), 3.28 (d, 2H), 1.81 (m, 1H), 1.50–1.20 (m, 8H), 1.00–0.85 (m, 6H).

**pH** (in CDCl_3_): δ 7.39 (d, 2H), 7.31 (d, 2H), 6.36 (s, 1H), 3.54 (s, SH), 3.22 (d, 2H), 1.70 (m, 1H), 1.45–1.25 (m, 8H), 0.91 (m, 6H).

**SA** (in DMSO-d): 7.60 (d, 2H), 7.49 (d, 2H), 6.12 (s, SH), 2.81 (m, 2H), 1.49 (m, 1H), 1.35–1.10 (m, 8H), 0.83 (t, 3H), 0.77 (t, 3H).

Preparation of **6a** and **7a**: 1 mol of TCTA was dissolved in acetone and 2 mol of Na_2_CO_3_ were added. Two moles of the respective amine were added slowly to the stirred mixture so that a temperature of 40 °C was not exceeded. After the addition the mixture was stirred for 5 h at 45 °C. The mixture was used without further purification for the next step.

Preparation of **8a**: 18.4 g (0.1 mol of TCTA was suspended at 10 °C in a 20 fold molar excess of ethanol (90 mL) and 0.1 mol (8.4 g) NaHCO_3_ were added. The mixture was stirred and slowly heated to 19 °C were CO_2_ formation began. The temperature was hold constant until no more gas was formed. Excess alcohol was eliminated under reduced pressure. Then, 0.1 mol bis(2-ethyl-hexyl)amine and 0.1 mol NaHCO_3_ were added and the mixture heated to 30 °C for 1 h. The oily product formed was used for the next step without any purification.

Preparation of **9a**: the procedure was analogue to the preparation of **6a** and **7a**, but instead of NaHCO_3_, Na_2_CO_3_ was used.

**6a**: 6-chloro-*N,N,N′,N′-*tetra(2-ethylhexyl)-1,3,5-triazine-2,4-diamine: ^1^H–NMR in ppm (CDCl_3_): 3.41 (d, 8H), 1.73 (m 4H), 1.26 (m, 32H), 0.87 (m, 24H). ^13^C-NMR: 168.9 (C–Cl, 1C), 165.6 (C-N, 2C), 50.1, 37.5, 30.8, 28.9, 24.1, 23.3, 14.3, 11.0. IR (cm^−1^): 2930, 1560, 1488, 1428, 1307, 1229, 1167, 971, 853.

**7a**: 6-chloro-*N,N,N′,N′-*tetra(n-butyl)-1,3,5-triazine-2,4-diamine: ^1^H–NMR in ppm (CDCl_3_): 3.49 (d, 8H), 1.55 (m 8H), 1.31 (m, 8H), 0.94 (m, 12H).

**8a**: 4-chloro-6-ethoxy-*N,N*-bis(2-ethylhexyl)-1,3,5-triazine-2-amine: ^1^H–NMR in ppm (CDCl_3_): 4.19 (c, 2H) 3.48 (m, 4H), 1.76 (m 2H), 1.48–1.11 (m, 19H), 0.88 (t, 12H).

**9a**: 2-chloro-4,6-bis((2-ethylhexyl)oxy)-1,3,5-triazine: ^1^H–NMR in ppm (CDCl_3_): 4.25 (m, 4H), 1.66 (m, 2H), 1.40–1.22 (m, 16H), 0.87 (t, 12H).

**10a**: 2-chloro-4,6-bis((2-ethylhexyl)thio)-1,3,5-triazine: ^1^H–NMR in ppm (CDCl_3_): 3.14 (d, 4H), 1.64 (m, 2H), 1.31 (m, 16H), 0.90 (t, 12H).

Substitution of the third chlorine of TCTA: to the obtained di-substituted TCTA solution, 1.03 mol thiourea was added and the mixture heated to 65 °C under a flux of nitrogen. After two hours, two mol of NaOH powder were added to the mixture.

**6b**: 6-mercapto-*N,N,N′,N′-*tetra(2-ethylhexyl)-1,3,5-triazine-2,4-diamine: ^1^H–NMR in ppm (CDCl_3_): 3.41 (d, 8H), 2.46 (d, 1SH), 1.73 (m 4H), 1.26 (m, 32H), 0.87 (m, 24H). IR (cm^−1^): 2931, 1593, 1534, 1456, 1430, 1374, 1290, 1142.

**7b**: 6-mercapto-*N,N,N′,N′-*tetra(n-butyl)-1,3,5-triazine-2,4-diamine: ^1^H–NMR in ppm (CDCl_3_): 3.48 (d, 8H), 2.49 (s, 1SH), 1.55 (m 8H), 1.32 (m, 8H), 0.94 (t, 12H).

**8b**: 4-mercapto-6-ethoxy-*N,N*-bis(2-ethylhexyl)-1,3,5-triazine-2-amine: ^1^H–NMR in ppm (CDCl_3_): 4.19 (c, 2H) 3.48 (m, 4H), 2.52 (d, 1HS), 1.76 (m 2H), 1.48–1.11 (m, 19H), 0.88 (t, 12H).

**9b**: 2-mercapto-4,6-bis((2-ethylhexyl)oxy)-1,3,5-triazine: ^1^H–NMR in ppm (CDCl_3_): 4.25 (m, 4H), 2.54 (d, 1SH), 1.66 (m, 2H), 1.40–1.22 (m, 16H), 0.87 (t, 12H).

**10b**: 2-mercapto-4,6-bis((2-ethylhexyl)thio)-1,3,5-triazine: ^1^H–NMR in ppm (CDCl_3_): 3.16 (d, 4H), 2.51 (s, 1HS), 1.64 (m, 2H), 1.31 (m, 16H), 0.89 (t, 12H).

Modification reaction of the polymer: The above mixture was poured without any purification into a sealable flask, the desired quantity of PVC was added, and the solution saturated with nitrogen. The reactor was then closed, heated to 85 °C and stirred for two hours. After this time the reactor contained only modified polymer dissolved in acetone and some NaCl which was filtered off. The dry, flexible polymer was obtained by casting off the acetone solution or by precipitation in methanol/water.

## 3. Results

It is well known that a very efficient and selective way to link molecules to the PVC chains is the nucleophilic substitution of its chlorine atoms using aromatic or hetero-aromatic thiol compounds [20].In what follows, we have tested different preparative approaches on how aromatic and hetero-aromatic thiols with aliphatic chains in ortho-, meta-, and/or para-position can be synthesized and linked to PVC in the most efficient manner. In particular, two synthetic strategies have been addressed, that are based on (a) substituted aromatic thiols and (b) on the use of trichlorotriazine (TCTA) as inexpensive starting material for the preparation of hetero-aromatic thiol compounds.

### 3.1. Use of Substituted Thiols

**Scheme 1:**
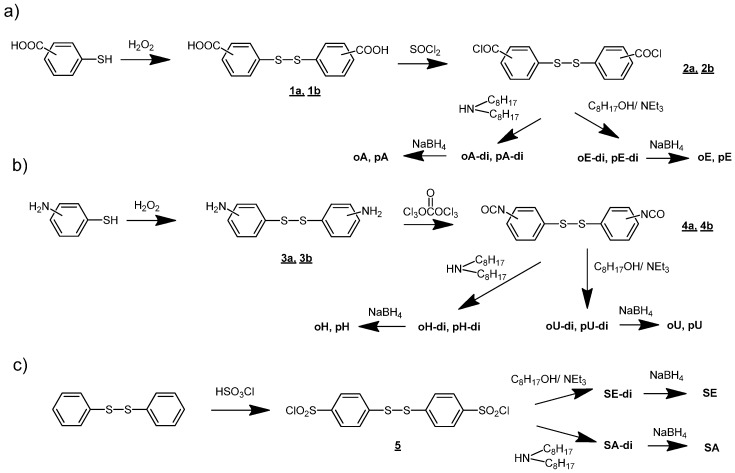
Synthesis of novel PVC-linkable modifiers.

**Scheme 2:**
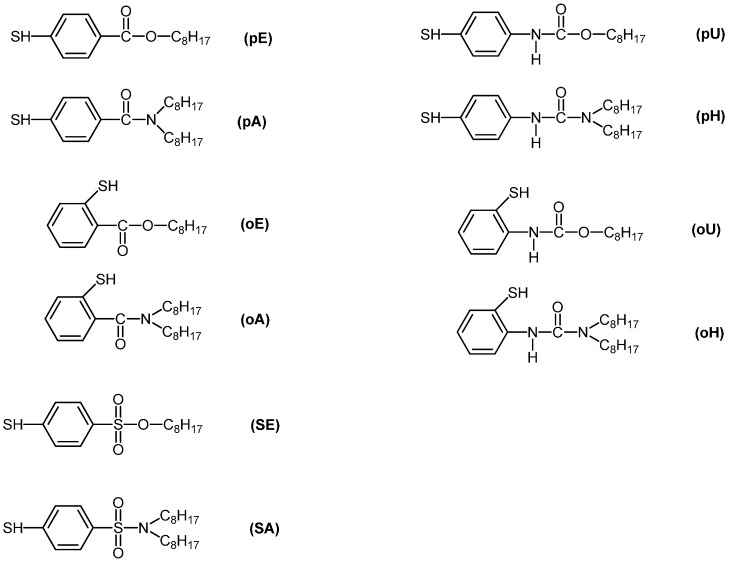
Structures of novel PVC-linkable modifiers.

There are several aromatic and hetero-aromatic thiols with functional groups commercially available. We have chosen as starting materials 2-mecaptobenzoic acid, 4-mercaptobenzoic acid, 2-aminothiophenol, and 4-aminothiophenol. Their reactivity towards aliphatic amines or alcohols, however, is limited and they have, therefore, to be activated. In order to avoid that the thiol group participates anyhow in the derivatization reaction of the functional group in ortho or para position we have, in a first step, protected the thiol group by oxidation forming the corresponding disulfides. In case **1a** and **1b** the carboxylic groups are then activated by transformation into the acid chlorides (**2a** and **2b**) by reaction with thionyl chloride. The amine containing disulfides (**3a** and **3b**), on the other hand, were activated using triphosgene, transforming them into the corresponding isocyanates **4a** and **4b**. (Scheme 1 and Scheme 2). It should be mentioned that, to our knowledge, the ortho-derivative **4a** has never been described before. In order to prepare an aromatic thiol compound carrying a chlorosulfonyl group (c) we started with commercial diphenyl disulfide which was subjected to a classical aromatic chlorosulfonation using chlorosulphonic acid. Product (**5**) is obtained with good yield (70%) as a slight yellow powder. The chlorosulfonyl groups are located exclusively in both para positions of the disulfide. This molecule was synthesized in the past by Bubert *et al*. [24] in a rather tedious four-step procedure with an overall yield of 15%.

In the second step, the five highly activated disulfides **2a**, **2b**, **4a**, **4b**, and **5** were reacted with an aliphatic alcohol (2-ethylhexylalcohol) or an aliphatic secondary amine (bis-(2-ethylhexyl)amine) obtaining the products where the aliphatic group is linked to the aromatic ring via an ester (**oE-di**, **pE-di**) or amide group (**oA-di**, **pA-di**), a urethane (**oU-di**, **pU-di**) or urea group (**oH-di**, **pH-di**) and a sulfonester (**SE-di**) or sulfonamide unit (**SA-di**).

The corresponding thiol compounds **oE**, **pE**, **oA**, **pA**, **oU**, **pU**, **oH**, **pH**, **SE**, and **SA** were finally obtained after deprotection of the disulfides by reduction with NaBH_4_. It is noteworthy that all compounds, with exception of **SE** (that required purification by column chromatography), could be used without any further purification for the final anchoring step to the polymer.

In order to facilitate and accelerate this modification reaction, the corresponding sodium thiolate salts were preformed using equimolar amounts of sodium hydroxide in ethanol. Other experimental conditions we have changed with respect to previous work [21] in order to force the reaction to complete conversion were the concentration, the solvent, and the temperature. Instead of carrying out the modification in cyclohexanone at 60 °C and at a concentration of 0.16 mol polymer per liter of solvent, in the present work we have used a mixture of acetone and THF (4:1), and the reactions were carried out at a concentration of 0.8 mol/L at 85 °C.

**Figure 1:**
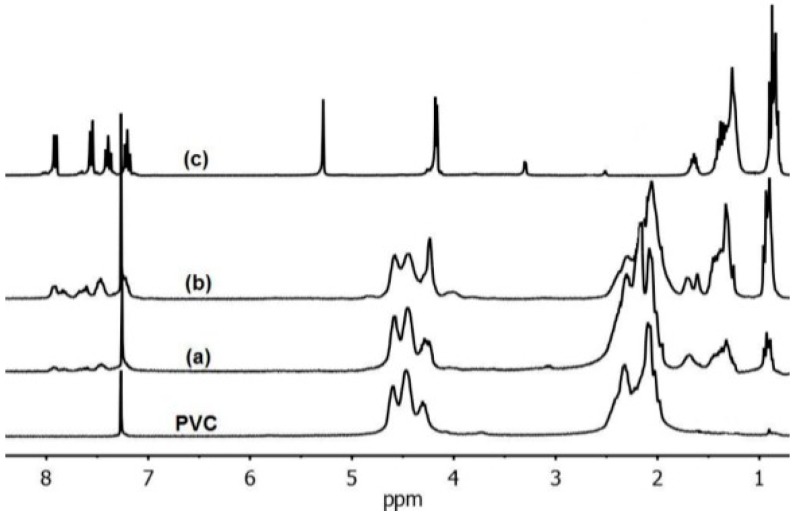
Series of ^1^H–NMR spectra of PVC modified with (**a**) 20 wt % and (**b**) 40 wt % of compound **oE**; and (**c**) shows the spectrum of pure **oE**.

In order to obtain polymers with varying degrees of modification we have reacted PVC with 20 and 40 wt % of the modifiers, respectively. After the anchoring reaction the modified polymers were purified by several precipitation/solution cycles in methanol/THF. The degrees of modification were determined from ^1^H–NMR spectra of the modified polymers. As an example the ^1^H–NMR spectrum of PVC modified with 20 and 40 wt % of **oE** is shown in Figure 1, where also the spectra of pure PVC and pure **oE** are depicted.

Apart from the broad PVC signals at 4.5 (CH–Cl) and 2.2 ppm (CH_2_), peaks corresponding to the modifier molecule have been formed in the aliphatic (between 0.9 and 1.8 ppm) and aromatic region (around 7.5 ppm) and at 4.2 ppm. Furthermore, there are new peaks in the area between 3 and 4 ppm that correspond to the newly-formed CH–S protons of the polymer chain. The reaction is highly selective with respect to chlorine substitution, while elimination does not take place, which is deduced from the colorless products and the absence of olefinic protons between 5 and 6 ppm.

The degree of modification “X” for this reaction is calculated using the integrals of the aliphatic proton peak of the modifier molecule at 0.9 ppm (corresponding to six protons) and that of the PVC chain at 4.5 ppm (a triplet which proceeds from CHCl protons of PVC, according to iso-, hetero-, and syndiotactic triads) and is given by:

*X* = *I*(0.9 ppm)/[6/(*I*(4.5ppm) – *I*(0.9ppm))] (1)

where *I*(*z*) corresponds to the intensity of the peak at *z* ppm.

The results obtained for the modification reactions with the other modifier molecules are calculated in an analogue manner and are summarized in Table 1. We have defined a degree of anchorage A as the relation of the nominal PVC/modifier amount and the degree of modification that was finally achieved.

As can be concluded from the results in Table 1, aromatic thiols to which the aliphatic chains are linked by an ester or amide group are highly reactive towards chlorine substitution on PVC and the degree of anchorage is around 95% in all of these cases, with the exception of **oE**. This means that in this way practically all modifier molecules in the system have been linked to the PVC chains. The results obtained for the compounds prepared via linkage to an isocyanate are somewhat less efficient with degrees of anchorage between 70% and 90%. This is most likely due to the strong electron withdrawing character of the urethane/urea bond that lowers the nucleophilicity of the thiol.

In Table 1 we have also listed the *T*g values of the obtained polymers in order to gather information concerning a future anchorage of plasticizers. In general, these are significantly lower than that of pure PVC (*T*g = 85 °C). However, as already shown in previous work on covalently-linked DOP [17], the plasticizer efficiency is limited when compared to systems in which the plasticizer is not bound to but physically dispersed in the polymer. This was explained by the increased rigidity of those chain segments to which the additive as bulky group is linked.

### 3.2. Trichlorotriazine-Based Thiols

An alternative way to prepare substituted thiols uses trichlorotriazine (TCTA) as an inexpensive starting material. This is an interesting and versatile substance as it has three chlorine atoms of different reactivities which allow for their step-wise selective substitution [25] by different nucleophiles when the appropriate reaction conditions are chosen.

We have used this chemistry to link two equivalents of aliphatic amines, thiols, and/or alcohols to the ring. Under optimized reaction conditions this reaction proceeds quantitatively when ethanol or/and aliphatic primary or secondary amines are used. Longer alcohols (>C2), methanol, and thiols, however, react only partially, secondary byproducts are formed and a purification procedure before substituting the third chlorine is required.

**Figure 2:**
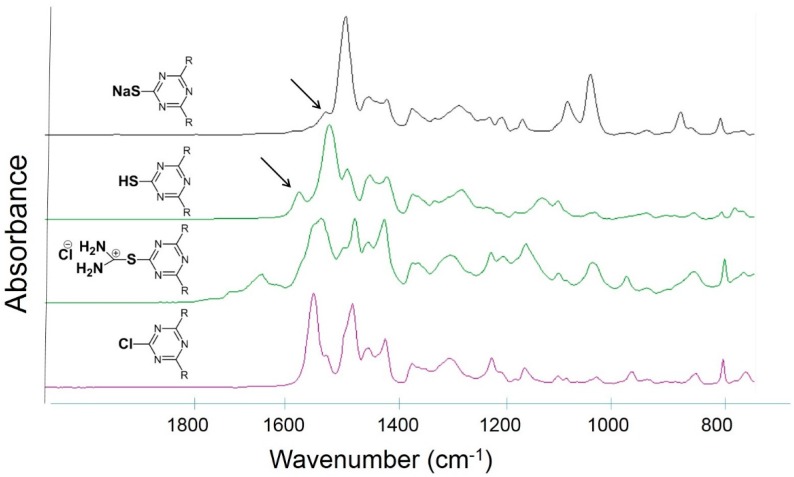
IR spectra of TCTA-derivatives of modifier **6** (see Table 2) being R = –N(C_8_H_17_)_2_.

A very interesting reaction is the transformation of this third chlorine into the mercapto compound using thiourea and the subsequent hydrolysis of the thiouronium salt formed as an intermediate. This step is completely quantitative and can be followed by IR spectroscopy (Figure 2).

**Figure 3:**
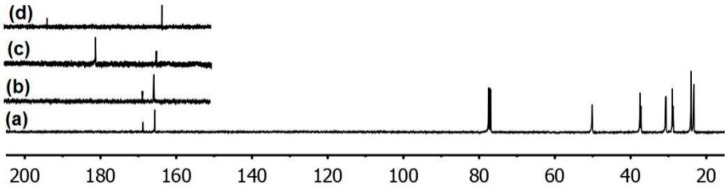
^13^C–NMR spectra of derivatives of modifier **6**: (**a**) TCTA-Cl; (**b**) TCTA-thiouronium salt; (**c**) TCTA-SH and (**d**) TCTA-SNa is 165.6 ppm. Similarly, the C–S shift of the thiouronium salt.

The salt is characterized by a broad band between 3500 and 2800 cm^−1^ and a peak at 1665 cm^−1^. This intermediate is easily hydrolyzed with aqueous ammonia solution or sodium hydroxide/ethanol to form the desired mercapto functionality. In these mercapto-containing TCTA compounds a tautomeric equilibrium between the thiol and the thione form exists which has in the past been studied by Stoyanov *et al.* [26] on structurally similar mercaptopyridines and mercaptopyrimidines. We have found that under ambient conditions the thione form is present in all mercapto TCTAs synthesized in this work. This is shown in Figure 3 where ^13^C–NMR spectra of the novel compounds are compared. In the chlorine containing derivatives the resonance shift of the hetero-aromatic ring C–Cl is 168.8 ppm and that of the two symmetric C–N carbons is at 168.1 ppm and that of the C–N carbon of these compounds at 165.1 ppm.

However, the thione structure of the mercapto compounds shows shifts at 164.7 ppm (C=S) and 180.8 ppm (C–N), respectively while in the sodium thiolate the aromatic character of the ring is restored again (193.5 and 164.4 ppm). The structural difference between thiol and thione derivatives is also confirmed by IR spectroscopy, where the intensities of the absorption bands in the range of the aromatic C–N bonds between 1500 and 1650 change significantly (Figure 2). In fact, the band at 1590 cm^−1^ of the thiol compound’s thione ring disappears completely in the corresponding thiolate salt.

**Scheme 3:**
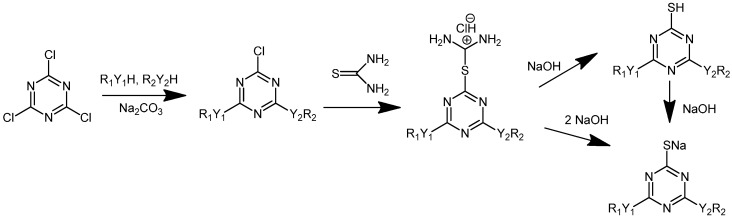
Synthesis of TCTA based PVC-linkable compounds. The structures of *Y* and *R* can be found in Table 2.

The formation and isolation of the mercapto-TCTA derivative may be avoided if two equivalents of NaOH in ethanol are used. In this way, the thiolate salt, which can be used without further purification for its reaction with PVC, is directly obtained. The complete synthetic route is summarized in Scheme 3.

A number of TCTA based sodium thiolates prepared using different types of nucleophiles (amines, alcohols, and thiols) with aliphatic chains have been synthesized and are summarized in Table 2. These compounds have been used to carry out modification reactions in acetone solutions of PVC using different amounts of modifier. For the anchoring step of the compounds to the polymer the same experimental conditions than those used in the first part of this work were used in order to force the reaction to complete conversion. That is, the modification reactions of the polymer were carried out in acetone at 85 °C and at a concentration of 0.8 mol polymer per liter of solvent.

**Figure 4:**
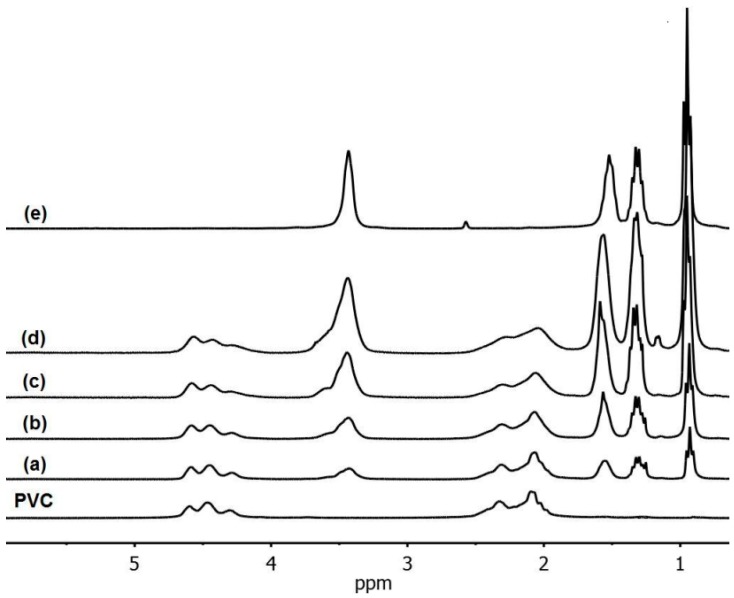
^1^H-NMR spectra of PVC modified with different amounts of compound **7**. (**a**) 6 mol %; (**b**) 11 mol %; (**c**) 22 mol%; (**d**) 34 mol%, and (**e**) pure compound **7**.

In order to analyze the modification reactions the obtained polymers were purified by several precipitation/solution cycles and the degrees of modification were determined from ^1^H-NMR spectra of the modified polymers. The results of these reactions are summarized in Table 2. As an example the ^1^H-NMR spectra of PVC modified with 6, 11, 22, and 34 mol % of **7** is shown in Figure 4 where also the spectra of pure PVC and pure **7** are depicted. Apart from the broad PVC signals at 4.5 (CH–Cl) and 2.2 ppm (CH_2_), peaks corresponding to the modifier molecule have been formed in the aliphatic region (between 0.9 and 1.6 ppm) and at 3.4 ppm. The intensity of the triplet type peak at 4.5 ppm, which proceeds from CHCl protons of PVC remains constant upon modification showing that new formed CH–S protons have their resonance signal in the same area what is in contrast to the modifications carried out with modifiers based on purely aromatic thiols (shown above) where newly-formed CH–S protons appear around 3.5. The reaction is highly selective with respect to chlorine substitution while elimination does not take place, even for the highest degrees of modification as can be stated from the absence of olefinic protons between 5 and 6 ppm and the white color of the obtained polymers.

The molar degree of modification “X” for this reaction is calculated using the integrals of the aliphatic proton peak of the modifier molecule at 0.9 ppm (corresponding to twelve protons) and that of the PVC chain at 4.5 ppm and is given by:

*X* = *I*(0.9 ppm)/12[*I*(4.5 ppm)] (2)

The results obtained for the modification reactions with the other compounds are calculated in an analogue manner and are summarized in Table 2 together with the degrees of anchorage of the different modifiers.

The most interesting result in Table 2 is the fact that all amine-substituted TCTA derivatives show degrees of anchorage higher than 95%, demonstrating that the modifiers prepared from aliphatic primary or secondary amines and/or ethanol as substituents are linked quantitatively to the polymer chains. This is particularly remarkable as the compounds have been prepared in a one-pot procedure and were used for the anchoring reaction to the polymer without being isolated or purified.

This clearly indicates that not only the chemical modification of the polymer is quantitative but, under the chosen experimental conditions, also the formation of the amine substituted TCTA, the formation of the thiouronium salt and its hydrolysis to the thiolate salt. On the other hand, the described procedure is less efficient when the modifiers are formed using aliphatic thiols or alcohols with longer chains. In these cases, the binding step of the nucleophile to TCTA is the limiting factor because secondary reactions take place to some extent, as is demonstrated by thin layer chromatography.

This synthetic pathway based on TCTA uses a new highly-efficient four-step process which is based on inexpensive starting materials like TCTA, thiourea, and aliphatic primary or secondary amines and can be carried out in a one-pot method, using stoechiometric amounts of agents. Due to the selectivity and completeness of all reaction steps, no purification of the final modifiers is needed and it can directly be used for its covalent linkage to the polymer. Under the chosen reaction conditions the binding step is also quantitative and selective as demonstrated by the absence of dehydrochlorination. All of these aspects make the process easily scalable and economically attractive. All previous attempts described in the literature (and addressed in the Introduction section) to prepare covalently-bound PVC-plasticizer systems were industrially non-viable, unlike this TCTA based chemistry. The procedure is also recommendable from an ecological point of view as only a minimum quantity of a non-toxic solvent, like acetone or methyl ethyl ketone (MEK), are required and only harmless byproducts like CO_2_, NaCl, and NH_3_ are formed. The TCTA-based route is also superior in terms of scalability with respect to the first route based on disulfides, which used relatively expensive aromatic thiols and chemical agents and lack the possibility of carrying out this multi-step synthesis as a one-pot reaction.

## 4. Conclusions

Two different synthetic strategies for the preparation of modifier molecules that allow for the covalent binding to PVC have been tested. The first one uses aromatic thiols that are protected as their disulfide. Flexible aliphatic chains with alcohols or amine functionality are then linked to the molecule via an activated functional group in ortho or para position of the rings. As functional highly-active groups’, like isocyanates, acidchlorides, or chlorosulfonyl, moieties were tested with the intention to achieve an easily-scalable process without the need of final purification. After deprotection by the reduction of the obtained compounds to the thiol, reactive modifier molecules are obtained. Due to the choice of a series of reactions that do generally proceed with excellent yields, no laborious purification of the product is necessary and overall high degrees of anchorage to the polymer are achieved, particularly if amide or ester functionalities are involved while the thiolates carrying urethane or urea bonds are somewhat less efficient due to a reduction of their nucleophilicity.

The second synthetic pathway towards linkable plasticizer molecules uses two of the three chlorine atoms of different reactivities of the trichlorotriazine (TCTA) to link flexible aliphatic chains with alcohols, amine, or thiol functionality, while the third chlorine is used to create the thiol via nucleophilic substitution by thiourea and subsequent hydrolysis. This synthetic route is green, economically feasible, and scalable since all reactions are quantitative and no purification is needed.

The presented approaches are highly versatile as different activations of aromatic and heteroaromatic rings are used. Therefore, many types of functional nucleophiles may be anchored to PVC providing non-migrating materials with a broad range of applications.

## Figures and Tables

**Table 1:** Results for the anchoring reaction of modifiers to PVC (*T*g of unmodified PVC: 85 °C).

Compound	Amount of modifier in the mixture with PVC (wt %)	Covalently bound modifier (wt %)	Degree of anchorage A (%)	*T*g (°C)
oE	20	14.4	72.0	55
	40	34.1	85.2	35
oA	20	18.5	92.5	56
	40	37.8	94.5	37
pE	20	18.7	93.5	51
	40	38.8	97.0	29
pA	20	18.6	93.0	53
	40	38.8	97.0	32
oU	20	13.9	69.5	54
	40	32.2	77.0	38
oH	20	14.8	74.0	62
	40	30.8	77.0	41
pU	20	13.9	69.5	53
	40	31.2	78.0	39
pH	20	17.2	86.1	60
	40	35.1	87.8	33
SE	20	-	-	-
	40	-	-	-
SA	20	15.1	75.5	49
	40	36.3	90.8	28

**Table 2:** Results for the anchoring reaction of modifier compounds to PVC.

	*Y*_1_	*R*_1_	*Y*_2_	*R*_2_	Modifier in the mixture (wt %)	Covalently bound modifier (wt %)	Degree of anchorage (%)	*T*g (°C)
6	N(C_8_H_17_)	C_8_H_17_	N(C_8_H_17_)	C_8_H_17_	20	19.5	97	42
					40	39.2	98	35
7	N(C_4_H_9_)	C_4_H_9_	N(C_4_H_9_)	C_4_H_9_	20	19.6	98	72
					40	39.6	98	55
8	O	C_2_H_5_	N(C_8_H_17_)	C_8_H_17_	20	19.9	98	43
					40	39.8	98	37
9	O	C_8_H_17_	O	C_8_H_17_	20	<8	-	-
					40	<10	-	-
10	S	C_6_H_13_	S	C_6_H_13_	20	<15	-	-
					40	<31	-	-
